# The prognostic role of functional dependency in older inpatients with COVID-19

**DOI:** 10.1186/s12877-021-02158-1

**Published:** 2021-03-31

**Authors:** Galina Plotnikov, Efraim Waizman, Irma Tzur, Alexander Yusupov, Yonatan Shapira, Oleg Gorelik

**Affiliations:** 1 Geriatric Assessment Unit, Yitzhak Shamir (Assaf Harofeh) Medical Center, Zerifin, Israel; 2 Sackler Faculty of Medicine, Tel Aviv University, Ramat Aviv, Israel; 3 Department of Internal Medicine “F“, Yitzhak Shamir (Assaf Harofeh) Medical Center, 7033001 Zerifin, Israel

**Keywords:** Functional status, ADL, Elderly, COVID-19, Hospitalization, Prognosis

## Abstract

**Background:**

Coronavirus disease 2019 (COVID-19) is a pandemic infection with substantial risk of death, especially in elderly persons. Information about the prognostic significance of functional status in older patients with COVID-19 is scarce.

**Methods:**

Demographic, clinical, laboratory and short-term mortality data were collected of 186 consecutive patients aged ≥ 65 years hospitalized with COVID-19. The data were compared between 4 study groups: (1) age 65–79 years without severe functional dependency; (2) age ≥ 80 years without severe functional dependency; (3) age 65–79 years with severe functional dependency; and (4) age ≥ 80 years with severe functional dependency. Multivariate logistic regressions were performed to evaluate the variables that were most significantly associated with mortality in the entire sample.

**Results:**

Statistically significant differences were observed between the groups in the proportions of males (*p* = 0.007); of patients with diabetes mellitus (*p* = 0.025), cerebrovascular disease (*p* < 0.001), renal failure (*p* = 0.003), dementia (*p* < 0.001), heart failure (*p* = 0.005), pressure sores (*p* < 0.001) and malignant disorders (*p* = 0.007); and of patients residing in nursing homes (*p* < 0.001). Compared to groups 1 (*n* = 69) and 2 (*n* = 28), patients in groups 3 (*n* = 32) and 4 (*n* = 57) presented with lower mean serum albumin levels on admission (*p* < 0.001), and were less often treated with convalescent plasma (*p* < 0.001), tocilizumab (*p* < 0.001) and remdesivir (*p* < 0.001). The overall mortality rate was 23.1 %. The mortality rate was higher in group 4 than in groups 1 − 3: 45.6 % vs. 8.7 %, 17.9% and 18.3 %, respectively (*p* < 0.001). On multivariate analysis, both age ≥ 80 years and severe functional dependency were among the variables most significantly associated with mortality in the entire cohort (odds ratio [OR] 4.83, 95 % confidence interval [CI] 1.88 − 12.40, *p* < 0.001 and OR 2.51, 95 % CI 1.02 − 6.15, *p* = 0.044, respectively). Age ≥ 80 years with severe functional dependency (group 4) remained one of the variables most significantly associated with mortality (OR 10.42, 95 % CI 3.27–33.24 and *p* < 0.001).

**Conclusions:**

Among patients with COVID-19, the association of severe functional dependency with mortality is stronger among those aged ≥ 80 years than aged 65–79 years. Assessment of functional status may contribute to decision making for care of older inpatients with COVID-19.

## Background

Coronavirus disease 2019 (COVID-19) is a pandemic infection caused by severe acute respiratory syndrome coronavirus 2 (SARS-CoV-2), with substantial risk of severe illness and death [1–5]. Age is the most important risk factor for death among patients with COVID-19 [1–5]. However, chronological age should not be used as a sole prognostic factor for decision making in the care of patients with COVID-19. In studies focused on older patients with COVID-19, additional factors such as male sex, certain comorbid conditions and various laboratory abnormalities were associated with increased mortality, similar to younger patients [6–9]. However, in elderly patient populations, certain specific conditions, such as dementia [7, 10], frailty [8, 9] and functional status [11, 12], have been reported to be associated with increased mortality risk.

Elderly populations are recognized as heterogeneous, due to variability in biologic age, functional status and comorbidities. Among older patients with COVID-19, the clinical profile and prognostic value of functional dependency according to age have not been reported. Therefore, the aim of this study was to compare demographic, clinical and laboratory characteristics, and short-term mortality among patients hospitalized for COVID-19, grouped according to age 65–79 and ≥ 80 years, with and without severe functional dependency.

## Methods

### Study population and design

The study was conducted as an observational single-center investigation. The eligible population comprised 430 consecutive adult patients, hospitalized with symptomatic COVID-19 in the corona facility of our tertiary university hospital during March–August 2020. The patients were admitted to the facility (capacity of 78 general and 12 intensive care beds) from the emergency department, or transferred from other departments. A diagnosis of COVID-19 was based on qualitative detection of SARS-CoV-2 RNA in a nasopharyngeal swab using the Allplex™ 2019-nCoV assay in a CFX96™ real-time polymerase chain reaction detection system. During the hospitalization, the patients were managed by attending physicians using the institutional standardized protocol for treatment of COVID-19, which was regularly modified according to current information from the medical literature. The study included only patients aged ≥ 65 years (*n* = 186). The endpoint of the investigation was the composite of all-cause death during the current hospitalization and 30 days following discharge.

The cohort was stratified according to age ≥ 80 years; the rationale for this cut-off was its selection in other relevant studies [2, 8, 12]. Functional status prior to admission was evaluated by the Katz Index of Independence in Activities of Daily Living (ADL) [13, 14]. Accordingly, functional dependency is rated dichotomously (dependent/independent) by the attribution of one point for each of six ADL functions: bathing, dressing, toileting, transfer, continence and feeding. Patients with and without severe functional dependency were defined according to the respective ADL scores 0–3 and 4–6. For analysis of associations of age and functional status with demographic, clinical and laboratory variables, and with mortality, patients were classified into four groups: (1) age 65–79 years without severe functional dependence (*n* = 69); (2) age ≥ 80 years without severe functional dependence (*n* = 28); (3) age 65–79 years with severe functional dependence (*n* = 32); and (4) age ≥ 80 years with severe functional dependence (*n* = 57).

Demographic, clinical and laboratory data were collected from electronic medical records. Pneumonia was defined as a new chest radiographic infiltrate, which was not due to another known cause. Renal failure was defined as estimated glomerular filtration rate < 60ml/min/1.73m^2^ at admission, using the Modification of Diet in Renal Disease equation [15]. Pressure sores were defined as injuries that break down skin and underlying tissue, and result from prolonged pressure on the skin. Vital status was registered according to information from hospital records and the registry of the Ministry of Internal Affairs.

### Statistical analysis

Categorical variables were described as frequencies and percentages. Continuous variables were evaluated for normal distribution using histograms, Q-Q plots and the Kolmogorov-Smirnov test. Normally distributed continuous variables were expressed as means and standard deviations, while skewed variables were reported as medians and interquartile ranges. Chi-square and Fisher’s exact tests were applied to compare categorical variables. Analysis of variance, the independent samples T-test, the Kruskal-Wallis test and the Mann-Whitney test were used to compare continuous variables. Statistical comparisons were performed between survivors and non-survivors, and between the four study groups. Multivariate logistic regressions were performed to evaluate the variables that most significantly associated with mortality in the entire cohort. The regression included two steps. In the first step, age category (≥ 80 vs. 65–79 years) and severe functional dependency (ADL scores 0–3 vs. 4–6) were analyzed while controlling for potential confounders: patient sex, comorbidities (hypertension, diabetes mellitus, cerebrovascular disease, renal failure, heart failure, obesity, coronary artery disease, pressure sores, chronic lung disease, malignant disorders and pneumonia), serum albumin and C-reactive protein (CRP) levels, and nursing-home residence. In the second step, the study group was forced into the regression with the potential confounders. The backward method was applied for elimination of variables using the Wald test and *p*-values > 0.1 as criteria for removal. All statistical tests were two sided and a *p*-value < 0.05 was considered statistically significant. Statistical analysis was performed using SPSS statistical software (IBM SPSS Statistics for Windows, version 24, IBM Corp., Armonk, NY, USA, 2016).

## Results

### Characteristics and outcomes of the entire sample

The demographic, clinical and laboratory characteristics of the 186 patients included in the study are presented in Table 1. The mean age was 78.5 ± 8.6 years; 43.0 % were males. The mortality rate was 23.1 %. Non-survivors were older and more likely to present with severe functional dependency, pneumonia, cerebrovascular disease, renal failure, dementia, heart failure and pressure sores than survived patients; and were more often nursing-home residents. On admission, patients who later succumbed demonstrated lower mean levels of serum albumin and higher median values of serum CRP. Compared to survivors, non-survivors were more often treated with antibiotics, oxygen and mechanical ventilation, and admitted to the intensive care unit. Groups 1–4 comprised 37.1 %, 15.1 %, 17.2 and 30.6 % of the patients, respectively.

**Table 1:** Characteristics of the patients included in the study, according to survival

Variable	Entire group (***n***=186)	Survivors (***n***=143)	Non-survivors (***n***=43)	*p-value
Age (years)	78.5±8.6	77.0±8.2	83.6±8.1	**<0.001**
Male sex	80 (43.0%)	58 (40.6%)	22 (51.2%)	0.22
*Comorbid conditions*				
Hypertension	154 (82.8%)	118 (83.7%)	36 (82.5%)	0.86
Diabetes mellitus	96 (51.6%)	69 (51.7%)	22 (51.2%)	0.96
Severe functional dependency	89 (47.8%)	33 (23.1%)	21 (48.8%)	**<0.001**
Cerebrovascular disease	86 (46.2%)	61 (42.7%)	25 (58.1%)	**0.007**
Renal failure	75 (40.3%)	47 (32.9%)	28 (65.1%)	**<0.001**
Dementia Nursing-home residence	74 (39.8%) 54 (29.0%)	16 (14.3%) 33 (23.1%)	27 (62.8%) 21 (48.8%)	**<0.001** **<0.001**
Heart failure	42 (22.6%)	25 (17.5%)	17 (39.5%)	**0.002**
Obesity	40 (21.5%)	29 (20.3%)	11 (25.6%)	0.46
Coronary artery disease	40 (21.5%)	29 (20.3%)	11 (25.6%)	0.46
Pessure sores	32 (17.2%)	20 (14.0%)	12 (27.9%)	**0.034**
Chronic lung disease	30 (16.1%)	23 (16.1%)	7 (16.3%)	0.97
Malignant disease	25 (13.4%)	18 (12.6%)	7 (16.3%)	0.53
*Laboratory data*				
Serum albumin on admission (normal 34-48 g/l)	34.1±5.2	35.2±5.0	30.7±4.2	**<0.001**
Serum CRP on admission (normal 0.3-5.0 mg/l)	50.9 (17.2-123.0)	40.0 (15.3-102.0)	90.2 (43.8-171.5)	**<0.001**
Pneumonia during hospitalization	120 (64.5%)	83 (58.0%)	37 (86.0%)	**<0.001**
*Treatment during hospitalization*				
Antibiotics	133 (71.5%)	93 (65.0%)	40 (93.0%)	**<0.001**
Corticosteroids	81 (43.5%)	57 (39.9%)	24 (55.8%)	0.064
Convalescent plasma	40 (21.5%)	30 (21.0%)	10 (23.3%)	0.75
Tocilizumab	39 (21.0%)	30 (21.0%)	9 (20.9%)	0.99
Remdesivir	31 (16.7%)	25 (17.5%)	6 (14.0%)	0.59
Hydroxychloroquine	27 (14.5%)	18 (12.6%)	9 (20.9%)	0.17
Oxygen via nasal canula/mask	75 (40.3%)	66 (46.2%)	9 (20.9%)	**<0.001**
High flow oxygen	17 (9.1%)	6 (4.2%)	11 (25.6%)	**<0.001**
Mechanical ventilation	22 (11.8%)	5 (3.5%)	17 (39.4%)	**<0.001**
Admission to the intensive care unit	34 (18.3%)	12 (8.4%)	22 (51.2%)	**<0.001**

Data are presented as means ± standard deviations or medians (interquartile range) or numbers (percentages) of presented cases.

* Difference between survivors and non-survivors. *CRP* C-reactive protein. Bold entries in the table idicate a *p*-value of < 0.05.

On multivariate logistic regression analysis, age category (≥ 80 vs. 65 − 79 years) and severe functional dependency (ADL scores 0 − 3 vs. 4 − 6) were among the variables most significantly associated with mortality in the entire cohort (odds ratio [OR] 4.83, 95 % confidence interval [CI] 1.88 − 12.40, *p* < 0.001 and OR 2.51, 95 % CI 1.02 − 6.15, *p* = 0.044, respectively).

### Characteristics and outcomes according to age and functional dependency groups

Table 2 presents the demographic, clinical, laboratory and mortality data for the four study groups. Statistically significant differences were observed between the groups in the proportions of males (*p* = 0.007); of patients with diabetes mellitus (*p* = 0.025), cerebrovascular disease (*p* < 0.001), renal failure (*p* = 0.003), dementia (*p* < 0.001), heart failure (*p* = 0.005), pressure sores (*p* < 0.001) and malignant disorders (*p* = 0.007); and of patients residing in nursing homes (*p* < 0.001). A statistically significant difference was found across the groups in mean levels of serum albumin on admission (*p* < 0.001): the values were lower in groups 3 and 4 than in groups 1 and 2. No statistically significant differences were shown between the groups in the median values of serum CRP on admission and in evidence of pneumonia during hospitalization. Statistically significant differences were observed between the groups in treatments with convalescent plasma (*p* < 0.001), tocilizumab (*p* < 0.001) and remdesivir (*p* < 0.001): patients in groups 1 and 2 were treated more often than were patients in groups 3 and 4. The mortality rate was higher in group 4 than groups 1–3: 45.6 % vs. 8.7 %, 17.9 and 18.3 %, respectively (*p* < 0.001).

**Table 2:** Characteristics of the patients included in the study, according to age and functional dependency

Variable	Group 1 (age 65-79 years without severe functional dependency, ***n***=69)	Group 2 (age ≥80 years without severe functional dependency, ***n***=28)	Group 3 (age 65-79 years with severe functional dependency, ***n***=32)	Group 4 (age ≥80 years with severe functional dependency, ***n***=57)	*p-value
Age (years)	71.1±3.8	83.7±2.8	73.2±4.1	87.9±5.3	**<0.001**^a,b,c,d,e,f^
Male sex	37 (53.6%)	7 (25.0%)	18 (56.3%)	18 (31.6%)	**0.007**^a,c,d,f^
*Comorbid conditions*					
Hypertension	55 (79.9%)	22 (78.6%)	31 (96.9%)	46 (80.7%)	0.14^b,d,f^
Diabetes mellitus	38 (55.1%)	11 (39.3%)	23 (71.9%)	24 (42.1%)	**0.025**^a,b,c,d,f^
Cerebrovascular disease	12 (17.4%)	6 (21.4%)	23 (71.9%)	45 (78.9%)	**<0.001**^b,c,d,e^
Renal failure	16 (23.2%)	15 (53.6%)	14 (43.8%)	30 (52.6%)	**0.003**^a,b,c^
Dementia	2 (2.9%)	1 (3.6%)	23 (71.9%)	48 (84.2%)	**<0.001**^b,c,d,e^
Nursing-home residence	1 (1.4%)	2 (7.1%)	18 (56.3%)	33 (57.9%)	**<0.001**^b,c,d,e^
Heart failure	7 (10.1%)	5 (17.9%)	11 (34.4%)	19 (33.3%)	**0.005**^b,c,d,e^
Obesity	16 (23.2%)	6 (21.4%)	9 (28.1%)	9 (15.8%)	0.56
Coronary artery disease	16 (23.2%)	5 (17.9%)	8 (25.0%)	11 (19.3%)	0.87
Pressure sores	1 (1.4%)	0 (0%)	7 (21.9%)	24 (42.1%)	**<0.001**^b,c,d,e,f^
Chronic lung disease	10 (14.5%)	6 (21.4%)	8 (25.0%)	6 (10.0%)	0.27
Malignant disease	3 (4.3%)	3 (10.7%)	8 (15.6%)	14 (24.6%)	**0.007**^c,e^
*Laboratory data*					
Serum albumin on admission (normal 34-48 g/l)	36.7±4.0	36.2±5.1	32.4±5.0	30.9±4.4	**<0.001**^b,c,d,e^
Serum CRP on admission (normal 0.3-5.0 mg/l)	53.5 (17.7-123.7)	34.9 (9.1-99.2)	58.7 (18.4-136.6)	52.6 (20.4-124.9)	0.42
Pneumonia during hospitalization	42 (60.9%)	17 (60.7%)	23 (71.9%)	38 (66.7%)	0.69
*Treatment during hospitalization*					
Antibiotics	44 (63.8%)	18 (64.3%)	24 (75.0%)	47 (82.5%)	0.098^c,e^
Corticosteroids	31 (44.9%)	11 (39.3%)	16 (50.0%)	23 (40.4%)	0.79
Convalescent plasma	27 (39.1%)	7 (25.0%)	4 (12.5%)	2 (3.5%)	**<0.001**^a,b,c,e^
Tocilizumab	23 (33.3%)	8 (28.6%)	6 (18.8%)	2 (3.5%)	**<0.001**^c,e,f^
Remdesivir	23 (33.3%)	7 (25.0%)	1 (3.1%)	0 (0%)	**<0.001**^b,c,d,e^
Hydroxychloroquine	8 (11.6%)	2 (7.1%)	5 (15.6%)	12 (21.1%)	0.32
Oxygen via nasal canula/mask	31 (44.9%)	8 (28.6%)	7 (21.9%)	29 (50.9%)	**0.001**^a,b,e,f^
High flow oxygen	3 (4.3%)	0 (0%)	5 (15.6%)	9 (15.8%)	0.067^d,e^
Mechanical ventilation	4 (5.8%)	4 (14.3%)	6 (18.8%)	8 (14.0%)	0.23^b^
Admission to the intensive care unit	8 (11.6%)	4 (14.3%)	10 (31.3%)	12 (21.1%)	0.10^b,d^
Mortality	6 (8.7%)	5 (17.9%)	6 (18.3%)	26 (45.6%)	**<0.001**^c,e,f^

Data are presented as means ± standard deviations or medians (interquartile range) or numbers (percentages) of presented cases. *CRP* C-reactive protein. *Difference between all groups; ^a^Statistically significant difference between groups 1 and 2; ^b^Statistically significant difference between groups 1 and 3; ^c^Statistically significant difference between groups 1 and 4; ^d^Statistically significant difference between groups 2 and 3; ^e^Statistically significant difference between groups 2 and 4; ^f^Statistically significant difference between groups 3 and 4.

Table 3 presents the data of the multivariate analysis after introducing the study group into the regression. The *p*-values express statistical difference of groups 2–4 compared to reference group 1, which served as the reference. It can be seen that age ≥ 80 years with severe functional dependency (group 4) remained the variable most significantly associated with mortality: OR 10.42, 95 % CI 3.27–33.24 and *p* < 0.001. Moreover, the mortality risk was significantly higher for patients in group 4 than in groups 2 (*p* = 0.034) and 3 (*p* = 0.002). Additional variables that were strongly associated with mortality were: male sex, pneumonia, heart failure and CRP level (for each 10 mg/l increment).

**Table 3:** The variables that were most significantly associated with mortality in a multivariate logistic regression analysis

Variable	***p***-value	Odds ratio	95% confidence interval
Group 1 (age 65−79 years, without severe functional dependency); the reference group	<0.001	−	−
Group 2 (age ≥ 80 years, without severe functional dependency)	0.20	2.63	0.60−11.42
Group 3 (age 65−79 years, with severe functional dependency)	0.58	1.46	0.38−5.59
Group 4 (age ≥ 80 years, with severe functional dependency)	<0.001	10.42	3.27−33.24
Pneumonia	0.022	3.92	1.22−12.54
Heart failure	0.028	2.84	1.12−7.21
Male sex	0.057	2.37	0.98−5.76
Serum C-reactive protein (for each 10 mg/l increment)	0.026	1.06	1.01−1.11
Renal failure	0.074	2.20	0.93−5.22

## Discussion

The main novelty of this investigation of patients hospitalized with COVID-19 aged ≥ 65 years was the demonstration that the prognostic significance of functional status was greater among those aged ≥ 80 years. Indeed, mortality was extremely high (45.6 %) among patients aged ≥ 80 years with severe functional dependency and was lower, in the range of 8.7–18.8 %, among same-aged patients without severe functional dependency and among younger patients. Moreover, on multivariate analysis, age ≥ 80 years with severe functional dependency remained the variable most strongly associated with mortality (OR 10.42 and 95 % CI 3.27–33.24). Age, male sex, functional dependency, pneumonia, heart failure and elevated CRP levels were also found to be associated with mortality risk, thus collaborating data from other studies of older patients with COVID-19 [6–9, 11, 12].

A few studies on older patients with COVID-19 have been reported associations of frailty [8, 9] and functional status [11, 12] with increased risk of mortality. We chose the Katz Index of Independence in ADL to investigate the prognostic significance of functional status, due to its simplicity and effectiveness in measuring functional ability [13, 14]. Notably, our older patient population was hospitalized in general wards of the corona facility of our tertiary hospital. Geriatric consultation was available, but the patients were managed by internists. Considering this clinical setting and retrospective collection of data, we believe that the Katz Index was appropriate for evaluation of functional dependency. In another study, comprising 108 older inpatients aged ≥ 65 years with COVID-19, functional disability was also assessed by the Katz Index and its score ≤ 5 points was strongly associated with one-month mortality [11]. Elsewhere, among 375 patients hospitalized for COVID-19, mean age 66 years, functional status was evaluated by the Barthel index, using a 100-point score [12]. Each 5-point decrement in the Barthel score was associated with a 13 % increased risk of in-hospital death. The design of our investigation differed from the above-mentioned studies. First, we grouped the patients according to ages 65–79 and ≥ 80 years. Second, we dichotomized functional status as severe disability, the Katz Index scores of 0–3 vs. 4–6. Finally, the outcome of the study was the composite of in-hospital death and 30-day mortality following discharge.

Detailed evaluation of demographic, clinical and laboratory data revealed several interesting findings in the present study. Patients with rather than without severe disability were more likely to have certain comorbidities, to be nursing-home residents and to present lower mean levels of serum albumin on admission. Moreover, treatments with certain medications differed between patients with and without severe functional dependency.

The most important novel aspect of the present investigation is the demonstration that severe disability was more strongly associated with mortality among inpatients with COVID-19 aged ≥ 80 years than aged 65–79 years. The association of functional status with increased risk of mortality among hospitalized patients with COVID-19 may be explained by a number of underlying pathophysiological mechanisms. The poor outcome in patients with severe disability does not seem to be related to a more severe inflammatory status and clinical presentation of COVID-19, because the development of pneumonia during hospitalization and median values of serum CRP did not differ between the study groups. Frailty may have contributed to increased mortality among the older patients with severe functional dependency. Notably, an association of frailty with short-term mortality has been reported among inpatients with COVID-19 [8, 9]. Indeed, in the present study, patients with severe disability had a higher burden of comorbidities than did those without. Moreover, increased mortality in patients with severe functional dependency may be explained by a high frequency of dementia in these patients. This possibility is supported by data from the medical literature regarding an association of dementia with decreased survival in patients with COVID-19 [7, 10]. In addition, each of the other comorbid conditions examined (cerebrovascular disorder, heart failure, pressure sores and malignant disease) were more prevalent among patients with than without severe disability, and may have influenced the risk of mortality.

The poor prognosis of severe functional dependency in elderly patients with COVID-19 may be related to impaired nutritional status and host defense. Malnutrition is a common geriatric problem and associated with functional disability [16–18]. Our finding of an association of severe disability with lower levels of serum albumin on hospital admission supports this proposition, and is in concordance with the published data. Malnutrition in older patients with COVID-19 may be caused by reduced dietary intake resulting from decreased appetite and feeding problems, and also by malabsorption, nutrient losses and increased metabolic demands [16, 18]. Weight loss, sarcopenia, and deficiencies of proteins, vitamins and other nutrients are common among older malnourished patients and can contribute to multiorgan dysfunction that leads to increased risk of death [16, 18]. Moreover, in older people, malnutrition is associated with impaired cell-mediated immunity, cytokine production and phagocytosis, as well as progressive dysfunction of the immune system, namely immunosenescence [18]. This results in diminished immune response and systemic recovery.

The treatment of COVID-19 may also have contributed to the high mortality observed among the elderly patients with severe functional disability. Treatments were not limited by costs of relevant medications, admittance to the intensive care unit or lack of medical personnel. However, modification of the COVID-19 treatment protocol during the study period may have affected mortality. Patients in the study groups were similarly treated with corticosteroids, hydroxychloroquine and mechanical ventilation. However, patients with severe functional dependency more often tended to be treated with antibiotics and were less often treated with convalescent plasma, tocilizumab and remdesivir. The less frequent administration of the latter medications to patients with severe functional disability may be explained by requests for written informed consent for these treatments, which were considered experimental. For a considerable proportion of patients with severe functional dependency, informed consent could not be obtained due to cognitive impairment.

Our study has a number of limitations. First, this was a single center study and the results may not be generalizable to other medical centers. Second, the study included a relatively small cohort of patients. This may have affected statistical power for comparisons of some relevant data. Third, due to the retrospective design, missing data may have affected the results.

## Conclusions

Among older inpatients with COVID-19, especially aged ≥ 80 years, severe functional disability is strongly associated with an increased risk of short-term mortality. Assessment of functional status may contribute to decision making for the care of older patients hospitalized with COVID-19. We hope that vaccinating against COVID-19 will reduce the extremely high mortality rate in elderly patients with severe functional dependency.

## Data Availability

The datasets used and/or analyzed during the current study are available from the corresponding author on reasonable request.

## Abbreviations

COVID-19: Coronavirus disease 2019
SARS-CoV-2: :Severe acute respiratory syndrome coronavirus 2
ADL: Activities of Daily Living
CRP: C-reactive protein
OR: Odds ratio
CI: Confidence interval
